# Multimodal Imaging of Nanocomposite Microspheres for Transcatheter Intra-Arterial Drug Delivery to Liver Tumors

**DOI:** 10.1038/srep29653

**Published:** 2016-07-13

**Authors:** Dong-Hyun Kim, Weiguo Li, Jeane Chen, Zhuoli Zhang, Richard M. Green, Sui Huang, Andrew C. Larson

**Affiliations:** 1 Department of Radiology, Northwestern University Feinberg School of Medicine, Chicago, IL, USA; 2 Robert H. Lurie Comprehensive Cancer Center, Chicago, IL, USA; 3 Division of Hepatology, Northwestern University Feinberg School of Medicine Chicago, IL, USA; 4 Department of Cell and Molecular Biology, Northwestern University Feinberg School of Medicine Chicago, IL, USA; 5 Department of Electrical Engineering and Computer Science, Evanston, IL, USA; 6 Department of Biomedical Engineering, Northwestern University, Chicago, IL, USA; 7 International Institute of Nanotechnology (IIN), Northwestern University, Evanston, IL, USA

## Abstract

A modern multi-functional drug carrier is critically needed to improve the efficacy of image-guided catheter-directed approaches for the treatment of hepatic malignancies. For this purpose, a nanocomposite microsphere platform was developed for selective intra-arterial transcatheter drug delivery to liver tumors. In our study, continuous microfluidic methods were used to fabricate drug-loaded multimodal MRI/CT visible microspheres that included both gold nanorods and magnetic clusters. The resulting hydrophilic, deformable, and non-aggregated microspheres were mono-disperse and roughly 25 um in size. Sustained drug release and strong MRI T_2_ and CT contrast effects were achieved with the embedded magnetic nano-clusters and radiopaque gold nanorods. The microspheres were successfully infused through catheters selectively placed within the hepatic artery in rodent models and subsequent distribution in the targeted liver tissues and hepatic tumors confirmed with MRI and CT imaging. These multimodal nanocomposite drug carriers should be ideal for selective intra-arterial catheter-directed administration to liver tumors while permitting MRI/CT visualization for patient-specific confirmation of tumor-targeted delivery.

Hepatocellular cancer (HCC) is the world’s second leading cause of cancer death^1^; the poor prognosis for HCC patients is typically complicated by concurrent liver cirrhosis. Surgical intervention, consisting of resection and transplantation, remains the only curative approach for the treatment of HCC. However, given advanced disease stage and/or the lack of available donors for transplant, most patients are not considered surgical candidates. Non-surgical, primarily palliative approaches include percutaneous ablation (RFA, cryo- or micro-wave ablation) and transcatheter arterial chemoembolization (TACE). For decades, TACE has been a mainstay for the treatment of HCC patients with intermediate-stage disease^2^,^3^. TACE relies upon differences in the blood supply between HCC and normal liver tissues. Hepatic arteries supply ~90% of blood flow to HCC but only ~25% of flow to normal liver^4^. TACE involves arterial infusion of chemotherapeutic drugs (doxorubicin, cisplatin, and/or mitomycin-C) emulsified within lipiodol (viscous embolic oil) and embolization of the parent vessel with gel-foam or beads^5^,^6^,^7^. However, recent data suggests that even with catheter-directed hepatic arterial infusion, the systemic exposure to chemotherapy agents following conventional TACE remains high; many patients still suffer from systemic side effects. The overall survival benefit afforded by current liver-directed transcatheter approaches remains relatively modest^8^,^9^,^10^. Recently, drug-eluting bead (DEB) TACE approaches were developed for DOX delivery via non-resorbable hydrophilic polymers intended to permit sustained drug release for extended exposure of tumor tissues; however, despite providing a potentially preferable safety profiles, DEB-TACE has not yet demonstrated significant improvements in treatment outcomes compared with conventional TACE using iodinated oil.

TACE requires intra-procedural imaging with x-ray-based digital subtraction angiography (DSA) for catheter guidance and monitoring of subsequent drug infusion and embolization procedures. There is an increasing interest to introduce magnetic resonance imaging (MRI) into the interventional radiology suite to offer the benefit of functional measurements to guide therapeutic endpoints^11^,^12^,^13^. However, current DEB technology, lipiodol oil and the gel foams used in clinical settings are visible with both imaging modalities; importantly, the delivery of DEBs is not directly monitored as these are not visible with x-ray or MRI modalities^14^.

A modern multi-functional drug carrier for image guided catheter-directed procedures is critically needed to improve therapeutic outcomes. Incorporation of imaging agents into the drug source itself (i.e., a radiopaque/magnetic microspheres) should offer several advantages over current embolization agents not visible with clinical imaging modalities^15^,^16^,^17^,^18^. Multimodal MRI/CT visible microspheres would permit direct visualization of these drug carriers and during the delivery the tumoricidal drugs. Monitoring the distribution of these drug-loaded microspheres is paramount to determine the success of a given procedure to permit physicians to either administer additional microspheres to achieve an optimal tumor dose or even reposition the catheter to ensure complete coverage of the targeted lesion. In this study, MRI/CT visible drug eluting nanocomposite microspheres containing multimodal imaging contrast agents (magnetic clusters and Au nanorods) were fabricated by adopting a microfluidic gelation process. The drug release profile and imaging properties of these MRI/CT visible nanocomposite microsphere drug carriers were first evaluated *in vitro and in vivo* studies in orthotopic HCC rat models demonstrated potential for MRI/CT detection of these nanocomposite microspheres following intra-arterial infusion procedures.

## Materials and Methods

### Chemicals

FeCl_3_, polyacrylic acid (PAA), sodium hydroxide, diethylene glycol (DEG), hydrogen tetrachloroaurate(III) trihydrate (HAuCl_4_∙3H_2_0, 99.9%), silver nitrate (AgNO_3_, 99%), L-ascorbic acid (AA; C_6_H_8_O_6_, ≥98%), sodium borohydrate (NaBH_4_, 98%) and CTAB (cetyltrimethylammonium bromide, 99%), sodium alginate, calcium chloride, span 80 and n’-hexadecane were used as purchased from Sigma-Aldrich (St. Louis, USA). Amine-PEG-thiol (MW 5K) was purchased from Laysan Bio (Arab, Al, USA). 6-methoxyethylamino numonafide (MEAN), demonstrated efficacy against HCC was selected as a model drug^19^,^20^,^21^. 3-(4,5-dimethylthiazol-2-yl)-2,5-diphenyltetrazolium bromide (MTT, Sigma, USA) and dimethylsulfoxide (DMSO, Amresco, USA) were used for MTT assays.

### Synthesis of Magnetic Clusters and Au nanorods

Magnetic clusters were synthesized with a high temperature hydrolysis reaction^22^,^23^. Briefly, a NaOH/DEG stock solution (NaOH (2 g) in diethylene glycol (DEG) (20 ml)) was prepared. The solution was heated to 120 °C for 30 min under nitrogen, cooled, and kept at 70 °C. A mixture of FeCl_3_ (0.4 mmol), polyacrylic acid (PAA) (4 mmol), and DEG (17 ml) was heated to 220 °C in a nitrogen atmosphere for 30 min with vigorous stirring to form a transparent solution. NaOH/DEG stock solution (2 ml) was injected into the hot mixture. The resulting mixture was further heated for 1 h. The final products were washed with a mixture of Milli-Q water and ethanol 3 times and re-dispersed in Milli-Q water. 10 mmol MEAN was loaded on the magnetic clusters by mixing in ethanol/H_2_O (1:1, v/v) mixture, centrifuge and drying adsorption processes. Au nanorods were synthesized with a seed mediated method^24^. For synthesizing Au seeds, 250 uL of an aqueous 0.01 M solution of HAuCl_4_∙3H_2_0 was added to 7.5 mL of a 0.1 M CTAB solution in a glass vial. The solutions were gently mixed by inversion. Next, 0.6 mL of an aqueous 0.01 M ice-cold NaBH_4_ solution was added, followed by rapid inversion mixing. The synthesized Au seeds were used for growing Au nanorods. 4.75 mL of 0.1 M CTAB, 200 uL of 0.01 M HAuCl_4_∙3H_2_0, and 30 uL of 0.01 M AgNO_3_ solutions were added, one by one, to a glass vial, followed by gentle mixing by inversion. 32 uL of 0.1 M AA was added to this mixture. Finally, 0.010 mL of the Au seed solution was added, and the reaction mixture gently mixed for 10 s before leaving undisturbed for at least 3 h. The synthesized Au nanorods were further modified with amine-PEG-thiol. 100 mL of aqueous amine-PEG-thiol (2 mM) was added to 10 mL of Au nanorods (0.02 M). The mixture was stirred and incubated at room temperature for 3 h to allow complete PEG modification of the Au nanorods with thiol of PEG. The mixture was then purified by centrifugation at 8500 rpm for 10 min. The supernatant was decanted, and the pellet was re- suspended in 5 mL MilliQ water.

### Microfluidic Fabrication of Nanocomposite Microspheres

Microfluidic chips with channel dimensions of 130 um in depth and width were fabricated by soft lithography techniques. A previously reported two-step droplet gelation process was adopted to produce nanocomposite microspheres^19^. An oil, used as the continuous phase, was composed of n’-hexadecane and span80 (2% w/w). An aqueous mixture solution of sodium alginate (2% w/w), MEAN-magnetic clusters (1.5% w/w) and Au nanorods (1.5% w/w) was prepared for the dispersed phase. All flow streams were individually driven using independent syringe pumps (New Era NE-1000, NY, USA) to maintain a constant flow rate. The inlet ports were connected to the syringes (20 ml) using Tygon tubing (.02 inch I.D). A microscope (Olympus, Japan) was used for observation. Microspheres generated by shearing the dispersed phase were collected in a calcium chloride (50 mM; 40 ml) reservoir. The resulting microspheres were separated from the oil solution with a centrifuge and magnetic separation procedure. These microspheres were subsequently washed 3 times with 30 mL of Milli-Q water.

### Characterization of Nanocomposite Microspheres

The morphology and size of the synthesized magnetic clusters and Au nanorods were characterized using a transmission electronic microscope (TEM; FEI Tecnai Spirit G2). The structures of the samples were determined by X-ray diffraction (XRD; D/MAX Rint2000, Rigaku, Japan) using Ni-filtered Cu-ka X-rays with the phase being identified by a comparison with the JCPDS database. The morphologies and magnetic properties of the synthesized microspheres were characterized with an optical microscope (CKX41, Olympus, Japan) equipped with a video camera (QColor5, Olympus, Japan), scanning electronic microscope (JEOL, JSM-5200, Japan), confocal laser-scanning microscope (Zeiss LSM 510 META laser scanning confocal, Germany) and superconducting quantum interface device (SQUID, MPMS-XL, USA). 300 microspheres within these optical images were randomly selected to estimate the average size and size distribution. Images were analyzed with the aid of image processing software (Image J).

### Drug Loading Efficiency and Release Studies

The amount of MEAN drug loaded into the microspheres during synthesis was measured by dissolving the fabricated nanocomposite microspheres in a 50 mM ethylenediaminetetraacetic acid (EDTA) solution with sonication for 10 min. Drug elution studies were performed to investigate MEAN release kinetics from nanocomposite microspheres incorporating alginate (40 wt%), magnetic clusters (30 wt%) and Au nanorods (30 wt%) at 37 °C. An aqueous sample solution (10 mg/ml; 1 ml) was placed in a membrane bag (Spectra/Por MWCO 10,000, Spectrum, Los Angeles, CA, USA) and then immersed in 40 ml of PBS (Phosphate Buffer Solution, pH 7.2) solution. The temperature of the medium was maintained at 37 °C using a water bath. At specific time intervals, PBS (1 ml) medium was extracted and replaced with fresh medium. The extracted PBS was diluted with ethanol (1:1 v/v) to measure MEAN concentrations. The concentration of released MEAN from each sample was determined using fluorescent spectroscopy (SpectraMax M5, Molecular Devices, CA, USA). These measurements were performed three times and averaged to determine percentages of cumulative drug release amounts over time. The MEAN loaded nanocomposite microspheres were also imaged using confocal fluorescent microscopy at characteristic excitation and emission wavelengths for MEAN (λ_ex_ = 445 nm and λ_em_ = 550 nm).

### *In vitro* Cytotoxicity of Nanocomposite Microsphere Drug-Delivery Platform

Cytotoxicity of the nanocomposite microspheres was evaluated with MTT assay. McA-RH7777 hepatoma cell line and Clone 9 liver cell line (ATCC, CRL-1601, Manassas, VA, USA) were cultured in respective Dulbecco’s Modified Eagle’s Medium and F-12K medium (ATCC, Manassas, VA, USA) supplemented with 10% fetal bovine serum (Sigma-Aldrich, MO, USA) and 0.1% gentamycin (Sigma-Aldrich, MO, USA). Cells from the exponential phase of the culture were harvested and diluted to a cell density of about 2 × 10^4^ per ml. 100 ul of the cell suspension was added to 180 ul of medium in each well of a 96-well plate, incubated at 37 °C, 5% CO_2_ and 95% air for 1 day. A 10 ul solution consisting of different amounts of nanocomposite microspheres (composed of 40 wt% alginate, 30 wt% magnetic clusters and 30 wt% Au nanorods but without MEAN loading as intention was to study biocompatibility of the drug carrier) was then added to the respective wells and incubated for specific periods of time. Control studies were conducted using dose wells with normal saline. Exposure time was 40 h. Treated cells were then rinsed with PBS before 20 ul of PBS containing 5 mg/ml of MTT was added prior to incubation for another 4 h. This was followed by the addition of 150 ul of DMSO and plate agitation for 10 min. The optical density (OD) of the contents in each well was then measured at 570 nm using a bioassay reader (SpectraMax M5, Molecular Devices, CA, USA). OD measurements were repeated in triplicate. Cell viability for each sample was calculated as the ratio between OD measurements within control and treatment wells (%viability = (OD_treatment_/OD_control_)*100). Significant differences were determined using the Student’s t-test where differences were considered significant (p < 0.05).

### Characterization of MR Relaxivity Properties

T_2_ relaxation times for the nanocomposite microspheres were determined using a 7 Tesla MRI scanner (Clinscan, Bruker, Billerica, MA, USA). Imaging phantoms were prepared by diluting samples in 1% agarose at various concentrations of nanocomposite microsphere. The atomic Fe concentrations of the stock solutions were determined using Inductively Coupled Plasma Spectroscopy (ICP-MS, Perkin Elmer, Waltham, MA, USA) and MRI signal changes were measured with increasing Fe concentrations of the nanocomposite microspheres. Samples were suspended within agarose phantoms. For T_2_ measurement, a Carr-Purcell-Meiboom-Gill (CPMG) sequence of 6 echoes was used with TR  =  1000 ms and TE  =  6.4–44.8 ms with an echo interval of 6.4 ms. The T_2_ values were calculated on a voxel-wise basis using a least squares single exponential fitting model and then a signal ROI selected for each phantom to report the mean T_2_ value. Finally, we calculated the linear fit line between these T_2_ relaxation values and nanocomposite microspheres concentration with corresponding slope thus providing relaxivity estimate (Origin 7.0, Northhampton, MA).

### Characterization of CT Attenuation Properties

CT imaging of agarose phantoms that included nanocomposite microspheres (concentrations ranging from 2 to 10 mg/ml) was performed using a Siemens SOMATOM Definition Flash (Siemens, Forchheim, Germany) with the following acquisition protocol: pitch 0.8, collimation 32 × 0.5 mm. 140 kVp, 260 mAs, FOV 96 × 114 mm, the matrix size 512 × 512. The data were reconstructed using a B40f kernel. CT images were analyzed using OsiriX (OsiriX Foundation, Geneva, Switzerland).

### McA-RH7777 Rat Hepatoma Model

McA-RH7777 hepatoma cells were implanted in the left lateral liver lobe during mini-laparotomy procedures in 10 male Sprague Dawley rats. Briefly, rats were anesthetized with isoflurane (mixture of 5% isoflurane and oxygen at 3 L/min). A mini-laparotomy performed and left hepatic lobe exposed. 1 × 10^6^ McA-RH7777 cells were injected in the left lateral lobe and surgical site closed in 2-layers. Tumors were allowed to grow for 7 days to reach a size >5 mm in diameter while observing animal daily for any signs of distress. All animal studies were performed in accordance with protocols approved by the Institutional Animal Care and Use Committee at Northwestern University.

### Hepatic Intra-arterial Transcatheter Infusion of Nanocomposite Microspheres

After 7 days of tumor growth, the following steps were used to invasively catheterize the left hepatic artery (LHA) for selective infusion of the nanocomposite microspheres in each animal^25^,^26^. First, rats were anesthetized with isoflurane induction. After laparotomy, a cotton-tipped applicator was used to expose the common hepatic artery (CHA), proper hepatic artery (PHA), and gastroduodenal artery (GDA). A micro bulldog clamp (World Precision Instruments, Sarasota, FL) was placed on the CHA to prevent bleeding during catheterization. 4-0 Vetacryl absorbable polyglycolic acid suture (Webster Veterinary, Devens, MA) was then used to ligate the GDA distally to control retrograde bleeding during catheterization. Next, a 24G SurFlash polyurethane catheter (Terumo Medical Co., Somerset, NJ) was inserted into the GDA, advanced into the PHA and then distally into the LHA. X-ray digital subtraction angiographic (DSA) was used to confirm catheter placement in common branch of PHA using iodinated contrast (Omnipaque, Amersham). After selective catheterization, 0.1 mL of heparin was infused before infusing the nanocomposite microspheres (20 mg in 250 ul of PBS); each infusion was followed by a 0.2 mL saline flush. The catheter was then withdrawn, and a 3-0 suture used to permanently ligate the GDA above the insertion position. Finally, abdomen was closed using two-layer technique. The animals were then moved to the MRI and CT scanner located adjacent to the surgical suite. Post MRI scans were typically performed 1 hour after IA infusion. This time delay was necessary to allow us to withdraw catheter, surgically close the animal, and move to adjacent MRI.

### MRI and CT Visualization of Nanocomposite Microsphere Delivery

MRI studies were performed using a Bruker 7.0T ClinScan high-field small animal MRI system with a commercial rat coil (Bruker Biospin). Body temperature was monitored continuously and controlled with a water-bed (SA Instruments, Stony Brook, NY). T_2_-weighted images were collected pre- and post-arterial infusion of the nanocomposite microspheres. MR scans were performed using a gradient-echo sequence with following parameters: TR/TE = 1,300/7.2 ms, 0.7 mm slice thickness, FOV 71 × 85 mm, 216 × 256 matrix, respiratory triggering with MRI-compatible small animal gating system (Model 1025, SA Instruments, Stony Brook, NY). *In vivo* contrast-to-noise ratio (CNR) was calculated as the ratio between the image contrast between tumor regions and liver regions with the image noise, which was given by CNR = (SNR_tumor rim_−SNR_liver_)/SNR_tumor rim_.

For *in vivo* CT imaging, a Siemens SOMATOM Definition Flash (Siemens, Forchheim, Germany) system was used. Rats (n = 6) were imaged post-infusion of the nanocomposite microspheres. Images were acquired using following parameters: 140 kVp, 260 mAs, 0.6 mm slice thickness, a matrix size of 512 × 512 and a 10 × 10 mm FOV. The data were reconstructed using a D30f kernel and analyzed using OsiriX (OsiriX Foundation, Geneva, Switzerland).

### Histology

Each rat was euthanized after catheterization and imaging procedures. HCC specimens were sliced at 2 mm intervals; these slices were further sectioned into 5 um thick slices for hematoxylin and eosin (H&E) and Prussian blue staining to identify regions of HCC tissue and deposition of the nanocomposite microspheres within the tumor. All slides were digitized at x200 optical magnification using a TissueFAXS microscope (TissueGnostics GmbH, Vienna, Austria). Post-processing was performed using the HistoQuest software package (TissueGnostics GmbH).

## Results

### Fabrication of Nanocomposite Microspheres

**Figure 1:**
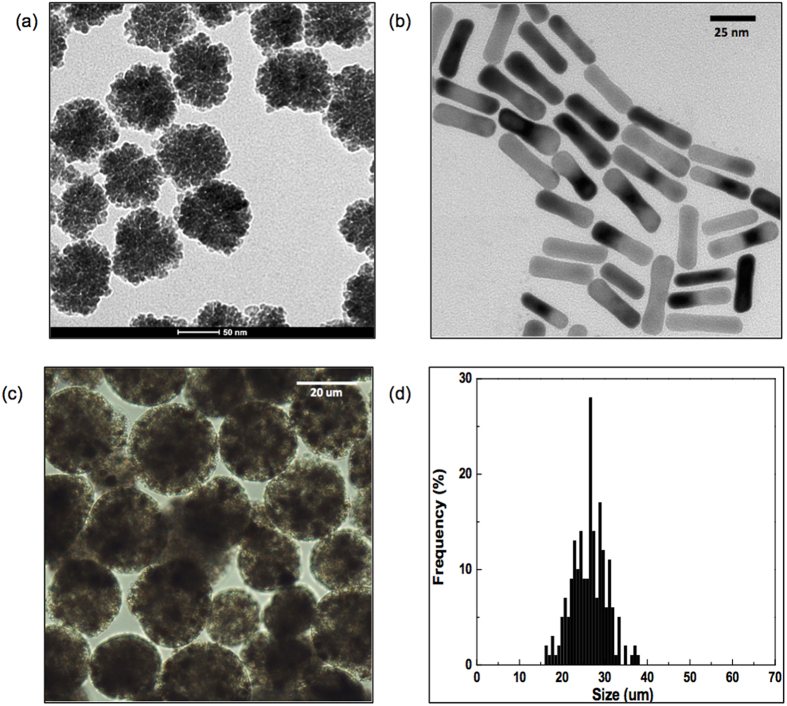
TEM images of (**a**) iron oxide nanoclusters and (**b**) gold nanorods, (**c**) optical microscopy image and (**d**) size distribution of microfluidic fabricated nanocomposite microspheres (incorporating alginate, iron oxide nanoclusters and Au nanorods).

**Figure 2:**
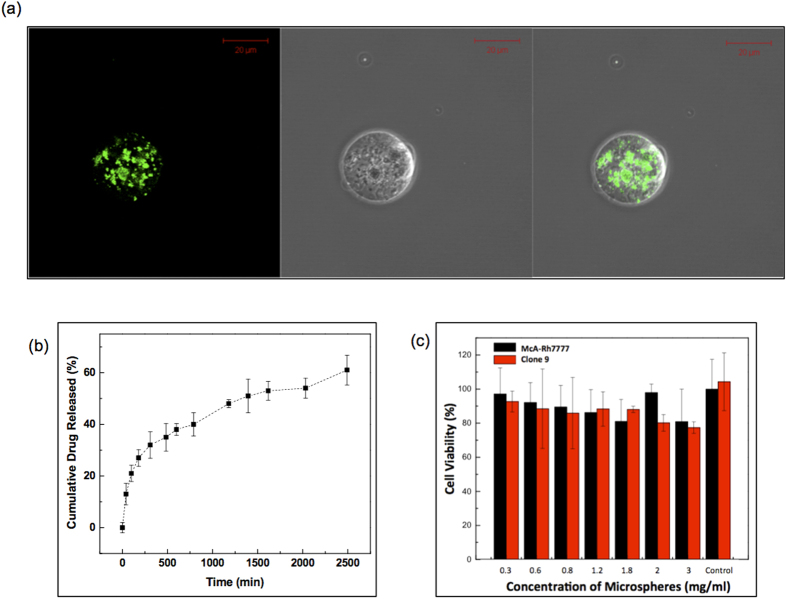
(**a**) Fluorescent microscope images of MEAN loaded nanocomposite microspheres (MEAN: green fluorescent (λ_excitation_ = 445 and λ_emission_ = 550 nm)), (**b**) MEAN drug release profile of nanocomposite microspheres and (**c**) cytotoxicity of the nanocomposite microspheres (alginate:magnetic nanoclusters:Au nanorods = 1.33:1:1 wt%) in McA-Rh7777 hepatoma cells and Clone9 liver epithelial cells.

~62 nm iron oxide nanoclusters (Fig. 1a) and Au nanorods with average dimensions (length × width) of 33 × 8.6 nm (Fig. 1b) were synthesized. The XRD patterns of the prepared magnetic clusters were indexed to the (220), (311), (400), (511), (440), (422), and (533) planes of a cubic unit cell (Fig. S1), which primarily corresponds to that of the magnetite structure (JCPDS No. 79-0417)^27^. These magnetic clusters were supramolecular structures formed by controlled agglomeration of iron oxide nanoparticles. The magnetic cluster solution was strongly water-dispersible and stable in aqueous solution with a surface charge of −42.1 mV (zeta-potential). 75% of initial amount of drug (MEAN) was loaded onto the magnetic clusters. Then, Au nanorods were synthesized with seed mediated methods. The synthesized Au nanorods also confirmed the fcc Au crystalline structure in the XRD measurement. The peak indexing were corresponding to (111), (200), (220) and (311) reflections of Au (Fig. S2). The length of the Au nanorods was 32 nm and the aspect ratio of the rods was 3.83. These MEAN-magnetic clusters and Au nanorods were incorporated into the alginate microsphere using a microfluidic channel. The microfluidic fabrication methods produced nanocomposite microspheres when using 5 ul/min and 25 ul/min flow rates for the dispersed and continuous oil phase solutions, respectively (Fig. 1c). The sheared microsphere aqueous phase in the microfluidic channel was gelled into solid nanocomposite microspheres upon contact with CaCl_2_ solution by crosslinking. The MEAN-magnetic clusters and Au nanorods were encapsulated within the microspheres (Fig. 1c). The shape of the microspheres remained spheroidal after the crosslinking gelation. The measured average diameter of the microspheres was 25 um with a variation of 10%, as demonstrated by the size distribution of these magnetic microspheres in aqueous solution (Fig. 1d). These nanocomposite microspheres demonstrated superparamagnetic properties preserving the magnetic properties of the magnetic clusters; incorporation of the Au nanorods was confirmed with a near infrared absorption at 800 nm (Fig. S1). Loading of the MEAN-magnetic clusters into the nanocomposite microspheres was confirmed with fluorescent images that showed the MEAN distribution within the nanocomposite microspheres (Fig. 2a). Drug-loaded nanocomposite microspheres demonstrated sustained MEAN release rates (47% in 24 hours) (Fig. 2b). The biocompatibility of these nanocomposite microspheres was validated during hepatoma and hepatic epithelial cell exposure studies (Fig. 2c). No significant toxicities were observed for either McA-Rh7777 hepatoma cells or the Clone 9 liver epithelial cell line upon exposure to these nanocomposite microspheres across concentrations ranging from 0 to 3 mg/mL.

### *In vitro* MRI and CT Imaging Characteristics of Nanocomposite Microspheres

**Figure 3:**
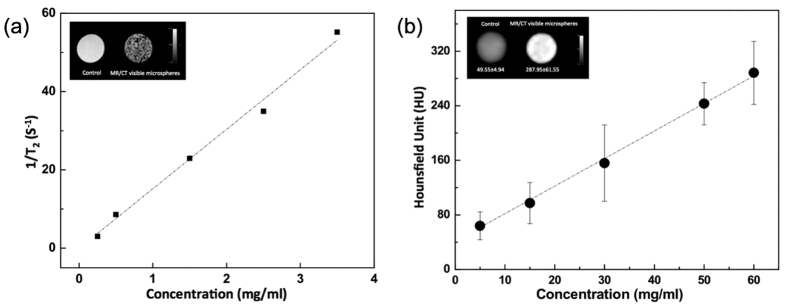
(**a**) A plot of R2 signal intensity versus nanocomposite microspheres concentrations in 1% agar phantoms and (inset) MRI T_2_-weighted images at TE = 32 ms showing the expected contrast effects by the nanocomposite microspheres in phantom and (**b**) Concentration versus CT signal (Hounsfield units, HU) curve of the nanocomposite microspheres. Inset shows CT images of the nanocomposite microspheres comparing with 1% agar phantoms.

Agar phantom signal intensities during T_2_-weighted MR imaging decreased with increasing concentration of nanocomposite microspheres encapsulating MEAN-magnetic clusters (1.5% w/w) and Au nanorods (1.5% w/w) (Fig. 3a). Across the five magnetic microsphere concentrations evaluated, T_2_-weighted signal decreased in proportion to increasing nanocomposite microsphere concentration. Thus, quantitative R2 (=1/T_2_) measurements increased with increasing microsphere concentration (Fig. 3a); the calculated r2 relaxivity from these measurements was 15.2 mg^−1^s^−1^. Next, the CT contrast characteristics of these microspheres were evaluated. Attenuation values, expressed in Hounsfield units (HU), were measured in agar phantoms with increasing concentration of the nanocomposite microspheres (5, 15, 30, 50 and 60 mg/mL). As demonstrated in Fig. 3b, as the concentration of nanocomposite microspheres increased, CT attenuation increased. HU as a function of microsphere concentration exhibited a linear relationship (R^2^ = 0.9917), described by the following equation: HU = 4.03651X + 41.529. (HU: CT numbers; X: concentration of nanocomposite microspheres, mg/mL) (Fig. 3b).

### Catheterization and MRI/CT Visualization of Nanocomposite Microsphere Delivery

**Figure 4:**
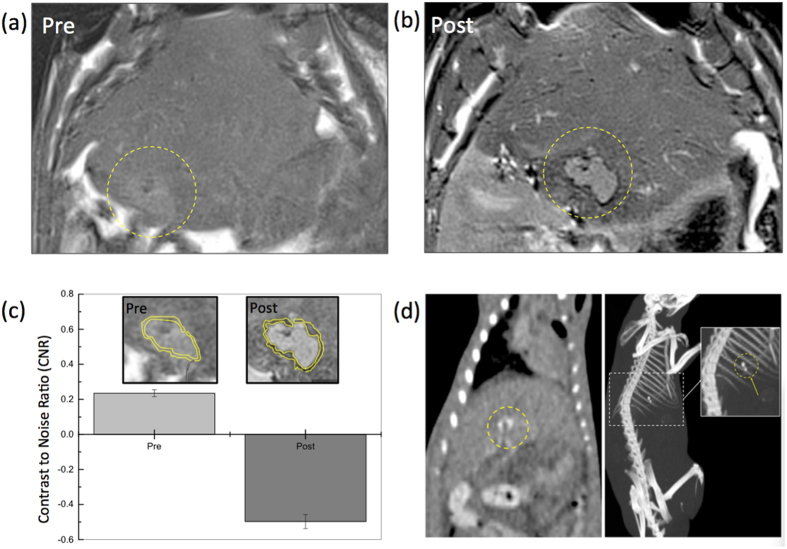
(**a**) T_2_-weighted MRI acquired (**a**) before or (**b**) after transcatheter intra-arterial infusion of nanocomposite microspheres in McA-RH7777 rat HCC models, (**c**) contrast to noise ratio (CNR) of tumor rim (inset: yellow roi regions) in MRI T_2_ weighted images at pre- and post-transcatheter infusion of nanocomposite microspheres, and (**d**) CT (left) coronal and (right) 3D MIP (maximum intensity projection) views (WL = 418, WW = 3106) of the whole mouse body after intra-arterial transcatheter infusion of nanocomposite microspheres in McA-RH7777 rat HCC models. Circles indicate regions with enhanced contrast.

Transcatheter intra-arterial infusion followed by *in vivo* MR and CT imaging of nanocomposites microsphere delivery was performed in HCC orthotopic rat model (Fig. 4). MRI was used to confirm tumor growth in each rat prior to catheterization procedures (Fig. 4a, Pre). Tumor sizes ranged from 2.5 to 8.1 mm in diameter 7 days post-implantation. Catheterization procedures were successfully performed in each animal. No adverse reactions or complications °Ccurred during or after the IA infusion procedures. Representative T_2_-weighted images acquired before and after transcatheter nanocomposite microsphere infusion are shown in Fig. 4a,b (and Fig. S3). Tumors were depicted as hyperintense relative to surrounding liver tissues in T_2_-weighted MRI images (Fig. 4a). Intra-hepatic microsphere delivery was restricted to the targeted tumor-bearing liver lobe with positions of nanocomposite microsphere deposition observed as local reductions to the signal intensity in the peripheral rim of tumor (Fig. 4b). To confirm the T_2_ contrast of the delivered nanocomposite microspheres, CNR of the rim area of tumor was compared at pre and post IA infusion of the nanocomposite microspheres. Significantly improved CNR of the tumor-rim area at post IA infusion was achieved by the nanocomposite microspheres in *in vivo* MRI (Fig. 4c). After MRI, the rats were imaged with CT to confirm the multi-modal visibility of these nanocomposite microspheres. CT coronal and 3D MIP (maximum intensity projection) views of a representative rat following microsphere infusion demonstrated enhanced attenuation (~205 HU) around the tumor position (Fig. 4b and Fig. S4).

### Histology

**Figure 5:**
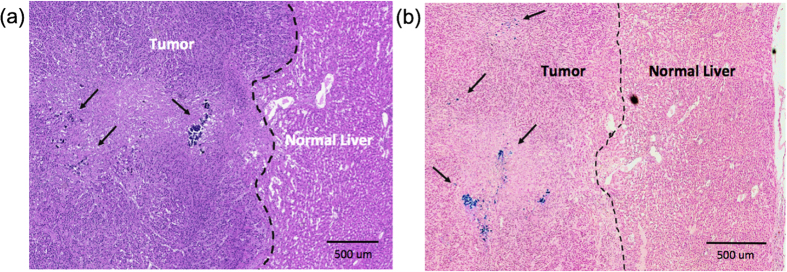
(**a**) H&E and (**b**) Prussian blue stained HCC liver tissues in McA-RH7777 rat hepatoma. Prussian blue staining of IA treated tumor tissues confirmed delivery of the nanocomposite microspheres (blue: microspheres deposits well depicted from representative histology slide). A region depicting both tumor rim and adjacent normal liver tissues from a treatment group.

Nanocomposite microsphere deposition within the peripheral rim of HCC tumor regions (as observed in MRI and CT images) was confirmed with H&E and Prussian blue staining in rat tissue specimens post-necropsy (Fig. 5a,b). The IA infused nanocomposites microspheres were preferentially distributed to blood vessels around the HCC tumor rim regions rather than the normal hepatic tissues.

## Discussion

Systemic chemotherapy is typically not well tolerated by liver cancer patients, particularly those with significant underlying hepatic dysfunction. A significant challenge for liver cancer chemotherapies is to deliver efficacious doses of drug to tumor tissues with minimal systemic toxicity. Another challenge is the highly variable biodistribution of chemotherapy drugs upon administration; patient-specific knowledge of the dose delivered to individual tumors should be valuable for timely adjustments to the therapeutic regimen. In our study, a nanocomposite microsphere drug-delivery platform was developed to permit transcatheter intra-arterial infusion and follow-up multi-modal (MRI/CT) imaging of the dose delivered to the targeted liver tumor tissues. Iron oxide nanoclusters and gold nanoparticles were incorporated into the fabrication of MRI/CT visible nanocomposite microspheres. The iron oxide nanoclusters demonstrated 3 times higher r_2_ relaxivity compared to non-clustered 7 nm iron oxide nanoparticles^19^,^23^. The hydrophilic PAA polymer and pores generated among the iron oxide nanoparticles increase interaction been susceptibility-induced local field gradients and hydrogen atoms of the local water molecules thus significantly increasing transverse relaxation rates. These strong T_2_ contrast effects permitted *in vivo* MRI detection of nanocluster microsphere-containing distribution upon catheter-directed infusion. Au nanoparticles have been widely used for CT imaging given a high X-ray absorption coefficient and the biocompatibility of Au nanoparticles^28^. In our study, Au nanorods were synthesized to provide a CT imaging component to our microspheres. MRI and CT imaging components and the chemotherapy drug MEAN were encapsulated within microspheres via microfluidic techniques. FDA approved biodegradable polysaccharide alginate polymers were selected for the microsphere matrix. Alginate importantly offers unique properties including biocompatibility, a relatively inert environment within the matrix, and a mild room temperature gelation process^19^,^29^. Microfluidic methods were effective for fabrication of nanocomposite microspheres encapsulating both imaging contrast nanoparticles and drug. The size of the nanocomposite microspheres was readily adjusted between 25 to 70 um by changing flow rates of dispersed and continuous phase channels. We targeted production of 20~40 um of nanocomposite microspheres considering diameter of the hepatic artery in rats^30^ thus ultimately producing 25 um nanocomposite microspheres (1.3:1:1 wt ratio of alginate, iron oxide nanoclusters and Au nanorods) with the following ratio of flow rates (5 ul/min: 25 ul/min, dispersed: continuous oil phase). MEAN drug was recently developed as a less toxic form of anti-neoplastic amonafide (DNA intercalater). MEAN has showed potent inhibition of HCC tumor cell growth with less toxicity than amonafide^19^,^20^. The chemotherapeutic agent (MEAN) loaded in the nanocomposite microspheres during the microfluidic process offers potent inhibition of tumor cell growth. A 75% drug loading efficiency was achieved along with sustained drug release characteristics (42% cumulative drug release within one day, drug release lasting for 2 weeks). However, when only the drug MEAN was loaded into the alginate microspheres (without inclusion of the iron-oxide nanoclusters), 90% MEAN drug was released within single day suggesting that the polyelectrolyte PAA-iron oxide nanoclusters slowed release rates, likely due to large pores among cross-linked alginate structures.

MR and CT imaging are the most important modalities for staging and follow-up imaging of hepatocellular carcinoma (HCC) following catheter directed chemotherapy^31^. Advantages of CT are that the modality is widely available, rapid, robust, and compared with MR imaging requires less expertise to perform and interpret acquired images. Disadvantages include radiation exposure and relatively low soft-tissue contrast. By comparison, MR imaging provides superior soft-tissue contrast and permits the assessment of a greater number of functional tissue properties, which in principle should assist in lesion detection and characterization. However, given potential ambiguities during MR imaging (eg. pre-existing signal voids prior to microsphere deposition), these MRI detection methods will likely require pre-infusion measurements and thus may be more time consuming and costly. Quantitative MRI methods typically require a greater level of expertise to perform and interpret images. Ultimately MRI and CT imaging are complementary for follow-up monitoring tumor response following catheter directed interventions for the treatment of HCC. The iron oxide cluster component of the microspheres demonstrated high r_2_ relaxivity (r_2_ = 199 mM^−1^s^−1^ at 3 T), which was higher MR r_2_ relaxivity than clinical grade Ferucarbotran (r_2_ = 146.1 mM^−1^s^−1^ at 3T), for strong MRI contrast effects^32^. At the same time, radio opaque Au nanorods were included for simultaneous CT contrast. Traditional CT imaging agents such as barium or iodine have high X-ray absorption coefficients but potentially serious renal toxicities. Gold nanoparticles are generally biocompatible and provide greater contrast than iodinated contrast agents due to the high atomic number (Z = 79) and k-edge value (80.7 keV)^33^. Furthermore, the contrast effects of iodine, which is sensitive to X-rays at low energies, is strongly dependent upon the environment^33^. For example, the X-ray attenuation of iodine in water is much lower than X-ray attenuation of iodine in air. The X-ray attenuation of gold nanoparticles is not significantly reduced in water. These results continue to suggest the strong potential for Au nanorods to serve as contrast agents for CT imaging^28^,^34^,^35^,^36^.

*In vivo* efficacy of the MRI/CT visible nanocomposite microspheres was evaluated following hepatic intra-arterial infusion procedure in McA-RH7777 rat hepatoma model. In our protocol, MRI was used for identifying tumor regions and MRI/CT used to confirm successful microspheres delivery to the targeted HCC following selective arterial infusion. For trans-arterial liver-directed therapies, visualization of the dose delivered to a targeted tumor could be used to adjust the administered dose (infuse additional drug-carrier) and/or adjust the position of the infusion catheter to achieve complete tumor coverage. Further, MRI/CT visualization of microsphere delivery should allow timely prediction of therapeutic outcome and patient prognosis.

## Conclusion

Microfluidics methods were used to fabricate multi-functional nanocomposite microspheres. The fabricated nanocomposite microspheres provided strong MRI T_2_ and CT image contrast and demonstrated sustained drug release behavior for a representative HCC-targeted drug (MEAN). The microspheres were successfully administered via hepatic intra-arterial routes. *In vivo* MRI/CT imaging was used to monitor intra-hepatic distribution and confirm delivery to the targeted tumor regions following catheter-directed infusion. These biocompatible MRI/CT visible nanocomposite microspheres should permit sustained targeted drug-delivery and valuable intra-procedural feedback for patient-specific optimization during liver-directed transcatheter therapies.

## Additional Information

**How to cite this article**: Kim, D.-H. *et al*. Multimodal Imaging of Nanocomposite Microspheres for Transcatheter Intra-Arterial Drug Delivery to Liver Tumors. *Sci. Rep.* **6**, 29653; doi: 10.1038/srep29653 (2016).

## Supplementary Material

Supplementary Information
