# Supplementary material for: Multimodal Imaging of Nanocomposite Microspheres for Transcatheter Intra-Arterial Drug Delivery to Liver Tumors
Source: Sci Rep. 2016 Jul 13;6:29653. doi: 10.1038/srep29653 (PMC4942792; doi:10.1038/srep29653)
Supplement: Supplementary Information [file srep29653-s1.pdf]

Supplementary Information

Multimodal Imaging of Nanocomposite Microspheres for Transcatheter Intra-Arterial Drug Delivery to Liver Tumors

Dong-Hyun Kim[a,b]*, Weiguo Li[a], Jeane Chen[a], Zhuoli Zhang[a,b], Richard M Green[c], Sui Huang[b], Andrew Larson[a,b,e,f,g]

[a]Department of Radiology, Northwestern University Feinberg School of Medicine, Chicago, IL, USA
[b]Robert H. Lurie Comprehensive Cancer Center, Chicago, IL, USA
[c]Department of Cell and Molecular Biology, Northwestern University Feinberg School of Medicine Chicago, IL, USA
[d]Division of Hepatology, Northwestern University Feinberg School of Medicine Chicago, IL, USA
[e]Department of Electrical Engineering and Computer Science, Evanston, IL, USA
[f]Department of Biomedical Engineering, Northwestern University, Chicago, IL, USA
[g]International Institute of Nanotechnology (IIN), Northwestern University, Evanston, IL, USA

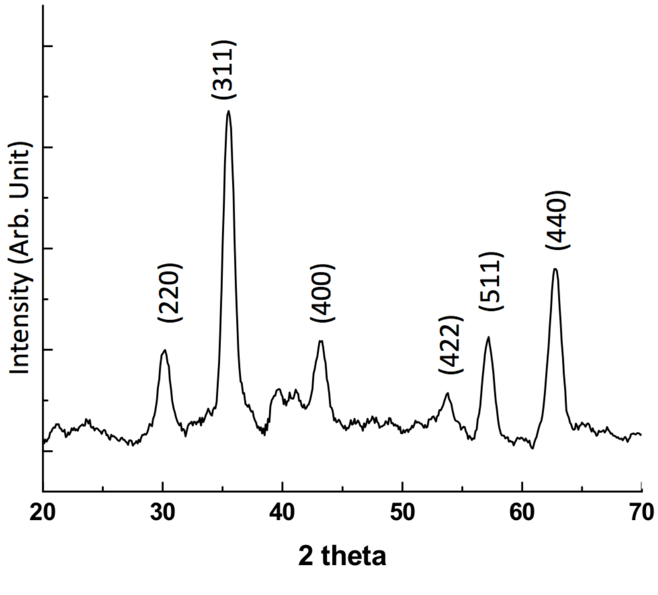

Figure S1. X-ray diffraction pattern of the synthesized magnetic clusters.

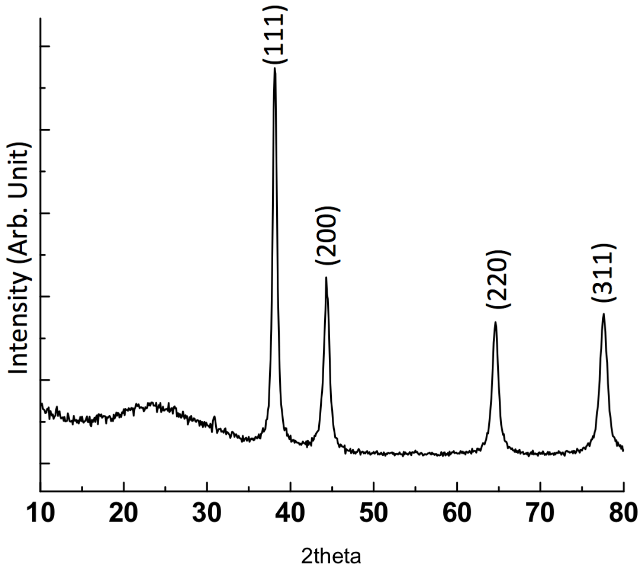

Figure S2. XRD pattern of the synthesized Au nanorods.

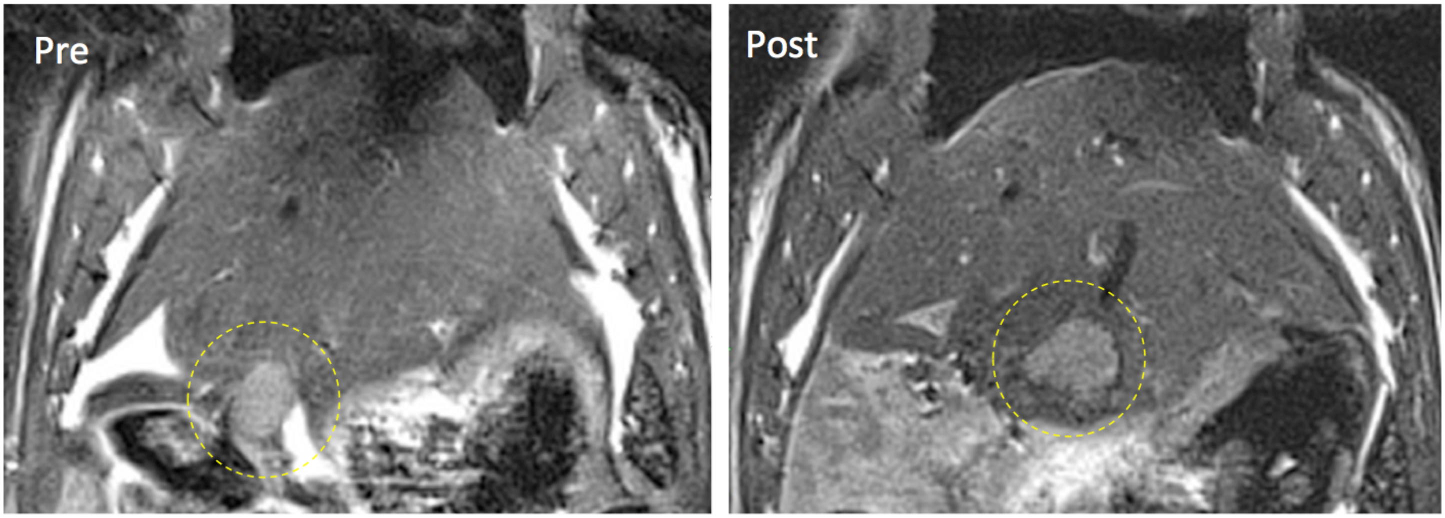

Figure S3. Additional T_2-weighted MRI acquired (a) before or (b) after transcatheter intra-arterial infusion of nanocomposite microspheres in McA-RH7777 rat HCC model. Note marked signal reduction at hypervascular tumor periphery upon intra-arterial delivery of the nanocomposite microspheres.

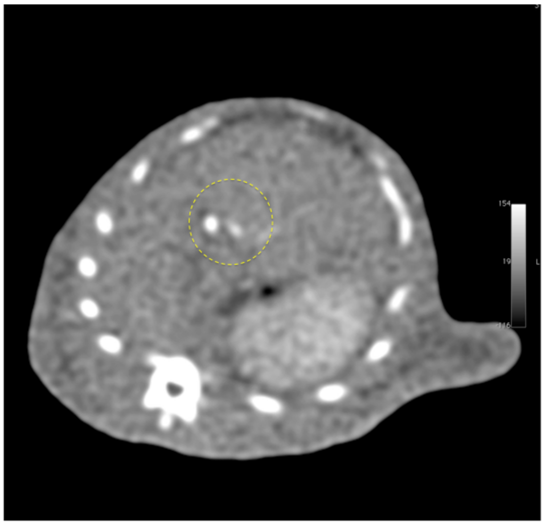

Figure S4. CT transversal image of rat after intra-arterial transcatheter infusion of nanocomposite microspheres in McA-RH7777 rat HCC models. Circles indicate regions with enhanced contrast within tumor region.
